# Supplementary material for: Nurse-assisted and multidisciplinary outpatient follow-up among patients with decompensated liver cirrhosis: A systematic review
Source: PLoS One. 2023 Feb 9;18(2):e0278545. doi: 10.1371/journal.pone.0278545 (PMC9910708; doi:10.1371/journal.pone.0278545)
Supplement: S1 Appendix — (PDF) [file pone.0278545.s001.pdf]

S1 appendix. Central search strategy

1	Liver cirrhosis
2	Cirrhosis
3	1 or 2
4	Nurs*
5	Nursing care
6	Nursing intervention
7	4 or 5 or 6
8	Patient
9	Outpatient
10	8 or 9
11	Rehabilitation
12	Aftercare
13	Follow-up
14	Outpatient care
15	11 or 12 or 13 OR 14
16	Post-discharge
17	Post-admission
18	Post hospitalization
19	16 or 17 or 18
20	3 AND
21	3 AND 7 AND 15
22	3 AND 7 AND 10 AND 15
23	3 AND 19
24	3 AND 19 AND 15

Full electronic search strategy for Pubmed:

1 Liver cirrhosis

2 Cirrhosis

3 1 or 2 ("liver cirrhosis"[MeSH Terms] OR ("liver"[All Fields] AND "cirrhosis"[All Fields]) OR "liver cirrhosis"[All Fields] OR ("liver cirrhosis"[MeSH Terms] OR ("liver"[All Fields] AND "cirrhosis"[All Fields]) OR "liver cirrhosis"[All Fields] OR "cirrhosis"[All Fields] OR "fibrosis"[MeSH Terms] OR "fibrosis"[All Fields]))

4 Nurs*

5 Nursing care

6 Nursing intervention

7 4 or 5 or 6 ("nurs*"[All Fields] OR ("nursing"[MeSH Subheading] OR "nursing"[All Fields] OR ("nursing"[All Fields] AND "care"[All Fields]) OR "nursing care"[All Fields] OR "nursing care"[MeSH Terms] OR ("nursing"[All Fields] AND "care"[All Fields])) OR (("nursing"[MeSH Terms] OR "nursing"[All Fields] OR "nursings"[All Fields] OR "nursing"[MeSH Subheading] OR "breast feeding"[MeSH Terms] OR "nursing s"[All Fields]) AND ("intervention s"[All Fields] OR "interventions"[All Fields] OR "interventive"[All Fields] OR "methods"[MeSH Terms] OR "methods"[All Fields] OR "intervention"[All Fields] OR "interventional"[All Fields])))

8 Patient

9 Outpatient

10 8 or 9 ("patient s"[All Fields] OR "patients"[MeSH Terms] OR "patients"[All Fields] OR "patient"[All Fields] OR "patients s"[All Fields] OR "outpatient s"[All Fields] OR "outpatients"[MeSH Terms] OR "outpatients"[All Fields] OR "outpatient"[All Fields])

11 Rehabilitation

12 Aftercare

13 Follow-up

14 Outpatient care

15 11 or 12 or 13 OR 14 "rehabilitant"[All Fields] OR "rehabilitants"[All Fields] OR "rehabilitate"[All Fields] OR "rehabilitated"[All Fields] OR "rehabilitates"[All Fields] OR "rehabilitating"[All Fields] OR "rehabilitation"[MeSH Terms] OR "rehabilitation"[All Fields] OR "rehabilitations"[All Fields] OR "rehabilitative"[All Fields] OR "rehabilitation"[MeSH Subheading] OR "rehabilitation s"[All Fields] OR "rehabilitational"[All Fields] OR "rehabilitator"[All Fields] OR "rehabilitators"[All Fields] OR ("aftercare"[MeSH Terms] OR "aftercare"[All Fields]) OR "follow-up"[All Fields] OR ("ambulatory

care"[MeSH Terms] OR ("ambulatory"[All Fields] AND "care"[All Fields]) OR "ambulatory care"[All Fields] OR ("outpatient"[All Fields] AND "care"[All Fields]) OR "outpatient care"[All Fields])

16 Post-discharge

17 Post-admission

18 Post hospitalization

19 16 or 17 or 18 (post-discharge"[All Fields] OR "post-admission"[All Fields] OR "post-hospitalization"[All Fields])

20 3 AND 7 (("liver cirrhosis"[MeSH Terms] OR ("liver"[All Fields] AND "cirrhosis"[All Fields]) OR "liver cirrhosis"[All Fields] OR ("liver cirrhosis"[MeSH Terms] OR ("liver"[All Fields] AND "cirrhosis"[All Fields]) OR "liver cirrhosis"[All Fields] OR "cirrhosis"[All Fields] OR "fibrosis"[MeSH Terms] OR "fibrosis"[All Fields])) AND ("patient s"[All Fields] OR "patients"[MeSH Terms] OR "patients"[All Fields] OR "patient"[All Fields] OR "patients s"[All Fields] OR ("outpatient s"[All Fields] OR "outpatients"[MeSH Terms] OR "outpatients"[All Fields] OR "outpatient"[All Fields])) AND ("nurs*"[All Fields] OR ("nursing"[MeSH Subheading] OR "nursing"[All Fields] OR ("nursing"[All Fields] AND "care"[All Fields]) OR "nursing care"[All Fields] OR "nursing care"[MeSH Terms] OR ("nursing"[All Fields] AND "care"[All Fields])) OR (("nursing"[MeSH Terms] OR "nursing"[All Fields] OR "nursings"[All Fields] OR "nursing"[MeSH Subheading] OR "nursing s"[All Fields]) AND ("intervention s"[All Fields] OR "interventions"[All Fields] OR "interventive"[All Fields] OR "methods"[MeSH Terms] OR "methods"[All Fields] OR "intervention"[All Fields] OR "interventional"[All Fields]))))

21 3 AND 7 AND 15 ("liver cirrhosis"[MeSH Terms] OR ("liver"[All Fields] AND "cirrhosis"[All Fields]) OR "liver cirrhosis"[All Fields] OR ("liver cirrhosis"[MeSH Terms] OR ("liver"[All Fields] AND "cirrhosis"[All Fields]) OR "liver cirrhosis"[All Fields] OR "cirrhosis"[All Fields] OR "fibrosis"[MeSH Terms] OR "fibrosis"[All Fields])) AND ("patient s"[All Fields] OR "patients"[MeSH Terms] OR "patients"[All Fields] OR "patient"[All Fields] OR "patients s"[All Fields] OR ("outpatient s"[All Fields] OR "outpatients"[MeSH Terms] OR "outpatients"[All Fields] OR "outpatient"[All Fields])) AND ("nurs*"[All Fields] OR ("nursing"[MeSH Subheading] OR "nursing"[All Fields] OR ("nursing"[All Fields] AND "care"[All Fields]) OR "nursing care"[All Fields] OR "nursing care"[MeSH Terms] OR ("nursing"[All Fields] AND "care"[All Fields])) OR (("nursing"[MeSH Terms] OR "nursing"[All Fields] OR "nursings"[All Fields] OR "nursing"[MeSH Subheading] OR "nursing s"[All Fields]) AND ("intervention s"[All Fields] OR "interventions"[All Fields] OR "interventive"[All Fields] OR "methods"[MeSH Terms] OR "methods"[All Fields] OR "intervention"[All Fields] OR "interventional"[All Fields]))) AND ("rehabilitant"[All Fields] OR "rehabilitants"[All Fields] OR "rehabilitate"[All Fields] OR "rehabilitated"[All Fields] OR "rehabilitates"[All Fields] OR "rehabilitating"[All Fields] OR "rehabilitation"[MeSH Terms] OR "rehabilitation"[All Fields] OR "rehabilitations"[All Fields] OR "rehabilitative"[All Fields] OR "rehabilitation"[MeSH Subheading] OR "rehabilitation s"[All Fields] OR "rehabilitational"[All Fields] OR "rehabilitator"[All Fields] OR "rehabilitators"[All Fields] OR ("aftercare"[MeSH Terms] OR "aftercare"[All Fields]) OR "follow-up"[All Fields])

22 3 AND 7 AND 10 AND 15 (("liver cirrhosis"[MeSH Terms] OR ("liver"[All Fields] AND "cirrhosis"[All Fields]) OR "liver cirrhosis"[All Fields] OR ("liver cirrhosis"[MeSH Terms] OR ("liver"[All Fields] AND "cirrhosis"[All Fields]) OR "liver cirrhosis"[All Fields] OR "cirrhosis"[All Fields] OR "fibrosis"[MeSH

Terms] OR "fibrosis"[All Fields])) AND ("patient s"[All Fields] OR "patients"[MeSH Terms] OR "patients"[All Fields] OR "patient"[All Fields] OR "patients s"[All Fields] OR ("outpatient s"[All Fields] OR "outpatients"[MeSH Terms] OR "outpatients"[All Fields] OR "outpatient"[All Fields])) AND ("nurs*"[All Fields] OR ("nursing"[MeSH Subheading] OR "nursing"[All Fields] OR ("nursing"[All Fields] AND "care"[All Fields]) OR "nursing care"[All Fields] OR "nursing care"[MeSH Terms] OR ("nursing"[All Fields] AND "care"[All Fields])) OR (("nursing"[MeSH Terms] OR "nursing"[All Fields] OR "nursings"[All Fields] AND ("intervention s"[All Fields] OR "interventions"[All Fields] OR "interventive"[All Fields] OR "methods"[MeSH Terms] OR "methods"[All Fields] OR "intervention"[All Fields] OR "interventional"[All Fields]))) AND ("rehabilitant"[All Fields] OR "rehabilitants"[All Fields] OR "rehabilitate"[All Fields] OR "rehabilitated"[All Fields] OR "rehabilitates"[All Fields] OR "rehabilitating"[All Fields] OR "rehabilitation"[MeSH Terms] OR "rehabilitation"[All Fields] OR "rehabilitations"[All Fields] OR "rehabilitative"[All Fields] OR "rehabilitation"[MeSH Subheading] OR "rehabilitation s"[All Fields] OR "rehabilitational"[All Fields] OR "rehabilitator"[All Fields] OR "rehabilitators"[All Fields] OR ("aftercare"[MeSH Terms] OR "aftercare"[All Fields]) OR "follow-up"[All Fields]))

23 3 AND 19 (("liver cirrhosis"[MeSH Terms] OR ("liver"[All Fields] AND "cirrhosis"[All Fields]) OR "liver cirrhosis"[All Fields] OR ("liver cirrhosis"[MeSH Terms] OR ("liver"[All Fields] AND "cirrhosis"[All Fields]) OR "liver cirrhosis"[All Fields] OR "cirrhosis"[All Fields] OR "fibrosis"[MeSH Terms] OR "fibrosis"[All Fields])) AND ("post-discharge"[All Fields] OR "post-admission"[All Fields] OR "post-hospitalization"[All Fields]))

24 3 AND 19 AND 15 (("liver cirrhosis"[MeSH Terms] OR ("liver"[All Fields] AND "cirrhosis"[All Fields]) OR "liver cirrhosis"[All Fields] OR ("liver cirrhosis"[MeSH Terms] OR ("liver"[All Fields] AND "cirrhosis"[All Fields]) OR "liver cirrhosis"[All Fields] OR "cirrhosis"[All Fields] OR "fibrosis"[MeSH Terms] OR "fibrosis"[All Fields])) AND ("post-discharge"[All Fields] OR "post-admission"[All Fields] OR "post-hospitalization"[All Fields]) AND ("rehabilitant"[All Fields] OR "rehabilitants"[All Fields] OR "rehabilitate"[All Fields] OR "rehabilitated"[All Fields] OR "rehabilitates"[All Fields] OR "rehabilitating"[All Fields] OR "rehabilitation"[MeSH Terms] OR "rehabilitation"[All Fields] OR "rehabilitations"[All Fields] OR "rehabilitative"[All Fields] OR "rehabilitation"[MeSH Subheading] OR "rehabilitation s"[All Fields] OR "rehabilitational"[All Fields] OR "rehabilitator"[All Fields] OR "rehabilitators"[All Fields] OR ("aftercare"[MeSH Terms] OR "aftercare"[All Fields]) OR "follow-up"[All Fields]))
