# Supplementary material for: Nurse-assisted and multidisciplinary outpatient follow-up among patients with decompensated liver cirrhosis: A systematic review
Source: PLoS One. 2023 Feb 9;18(2):e0278545. doi: 10.1371/journal.pone.0278545 (PMC9910708; doi:10.1371/journal.pone.0278545)
Supplement: S2 Appendix — (PDF) [file pone.0278545.s002.pdf]

S2 Appendix. Excluded studies and the reason for exclusion

Study	Study design	No of patients	Intervention	Reason for exclusion
**Case control studies**				
Tapper EB, et.al. A Quality Improvement Initiative Reduces 30-Day Rate of Readmission for Patients With Cirrhosis. Clinical gastroenterology and hepatology. 2016;14(5):753-9.	Prospective pre-post intervention with historic control	824	Adjustment of medical treatment	Only physician driven intervention on medical treatment. No specific nursing tasks.
**Clinical studies**				
Beg S et.al. Patient education and its effect on self-management in cirrhosis: a pilot study. European journal of gastroenterology & hepatology. 2016;28(5):582-7.	Prospective cohort	39	Leaflet and telephone interviews	No control-group. No specific nursing tasks
Thomson M et.al. An Automated Telephone Monitoring System to Identify Patients with Cirrhosis at Risk of Re-hospitalization. Digestive Diseases & Sciences. 2015;60(12):3563-9.	Prospective cohort	100	Telephone interviews	No control-group. No specific nursing tasks
Piano S et al. Predictors of Early Readmission in Patients With Cirrhosis After the Resolution of Bacterial Infections. The American journal of gastroenterology. 2017;112(10):1575-83.	Prospective follow-up	199	Standard treatment	No control-group. No specific nursing tasks
Alvarez MAet.al. Long-term clinical course of decompensated alcoholic cirrhosis: a prospective study of 165 patients. Journal of clinical gastroenterology. 2011;45(10):906-11.	Prospective cohort	165	Standard treatment	No control-group. No specific nursing tasks
**Qualitative studies**				
Lau-Walker M et.al. Patients with alcohol-related liver disease--beliefs about their illness and factors that influence their self-management. Journal of advanced nursing. 2016;72(1):173-85.	Cross-sectional	159	Interviews on illness beliefs, self-efficacy and emotional state	No control-group. No defined measures of intervention
Shabanloei R et.al. Stigma in Cirrhotic Patients: A Qualitative Study. Gastroenterology nursing : the official journal of the Society of Gastroenterology Nurses and Associates. 2016;39(3):216-26.	Consecutive enrolment	15	Interview on stigmas of cirrhosis	No control-group. No defined measures of intervention
Fagerstrom C, Frisman GH. Living with Liver Cirrhosis: A Vulnerable Life. Gastroenterology nursing : the official journal of the Society of Gastroenterology Nurses and Associates. 2017;40(1):38-46.	Consecutive enrolment	13	Interviews on vulnerability and reflection on life	No control-group. No follow-up measures of intervention.

Mikkelsen MR et .al. Alcoholic liver disease patients' perspective of a coping and physical activity-oriented rehabilitation intervention after hepatic encephalopathy. Journal of clinical nursing. 2016;25(17-18):2457-67.	Prospective	10	Interview on coping mechanisms	No control-group. All patients were included in Andersen 2013 (39)
**Retrospective studies**				
Seraj SM et.al. Hospital readmissions in decompensated cirrhotics: Factors pointing toward a prevention strategy. World journal of gastroenterology. 2017;23(37):6868-76.	Retrospective	132	Standard treatment	Report on readmission rates in cirrhosis, no intervention
Volk ML et.al. Hospital readmissions among patients with decompensated cirrhosis. The American journal of gastroenterology. 2012;107(2):247-52.	Retrospective	403	Standard treatment	Report on readmission rates in cirrhosis, no intervention
Bajaj JS et.al. The 3-month readmission rate remains unacceptably high in a large North American cohort of patients with cirrhosis. Hepatology (Baltimore, Md). 2016;64(1):200-8.	Retrospective report from prospective database	1353	Standard treatment	Admission rates and casual factors. No defined intervention
Lau Sy et.al. Hospital utilization in patients with liver cirrhosis after enrolment to the chronic liver failure program. Journal of Gastroenterology and Hepatology (Australia). 2014;29:95.	Retrospective follow-up	60	Standard treatment	No control-group.
**Reviews on outpatient management in cirrhosis**				
Mathews RE et.al. Outpatient management of cirrhosis: a narrative review. Southern medical journal. 2006;99(6):600-6	Narrative review		Evaluation of Standard treatment	No specific nursing tasks discussed.
Tapper EB. Building Effective Quality Improvement Programs for Liver Disease: A Systematic Review of Quality Improvement Initiatives. Clinical gastroenterology and hepatology. 2016;14(9):1256-65.e3.	Systematic review		Evaluation of Quality improvement programs	No specific nursing tasks discussed.
