# Supplementary material for: Nurse-assisted and multidisciplinary outpatient follow-up among patients with decompensated liver cirrhosis: A systematic review
Source: PLoS One. 2023 Feb 9;18(2):e0278545. doi: 10.1371/journal.pone.0278545 (PMC9910708; doi:10.1371/journal.pone.0278545)
Supplement: Attachment — Submitted filename: Response to Reviewers.docx [file pone.0278545.s004.docx]

Dear Academic Editor and Reviewers.

Thank you for your comments and recommendations.

We have listed your comments below in blue text and responded in black to each of the points raised. We have referred to the page and line for each change in the manuscript. Furthermore, we have attached a marked-up copy of the manuscript, with the changes made based on your comments and an unmarked version.

Best regards

Malene Barfod O’Connell

**Journal Requirements:**

When submitting your revision, we need you to address these additional requirements.

1. Please ensure that your manuscript meets PLOS ONE's style requirements, including those for file naming. The PLOS ONE style templates can be found at
   <https://journals.plos.org/plosone/s/file?id=wjVg/PLOSOne_formatting_sample_main_body.pdf> and 
   <https://journals.plos.org/plosone/s/file?id=ba62/PLOSOne_formatting_sample_title_authors_affiliations.pdf>

Response: The style requirements have been checked and followed.

2. Please upload a new copy of Figures 2 and 3 as the detail is not clear. Please follow the link for more information: <https://blogs.plos.org/plos/2019/06/looking-good-tips-for-creating-your-plos-figures-graphics/>" <https://blogs.plos.org/plos/2019/06/looking-good-tips-for-creating-your-plos-figures-graphics/>

Response: The figures have been changed into the format suggested in the PLOS ONE guideline.

**Reviewer #1:**

Patients with decompensated liver cirrhosis develop a number of complications that result in high morbidity and mortality and frequent readmission, require constant and rigorous monitor both in and outside the hospital. Nursing care to both hospitalized and nonhospitalized patients is most important to help manage and prevent complications of the disease and improve quality of life by providing medical education to patients and caregivers.

This comprehensive systemic review provide the valuable clinical practice evidence about the effect of nurses assisted outpatient follow up and multidisciplinary interventions. So I am pleased to recommed the editor to accept this paper.

**Response:** Thank you for the positive comments and recommendation.

**Reviewer #2:**

Here, authors determined the effects of nurse-assisted follow-up after admission with decompensation in patients with liver cirrhosis from all causes. Overall, the study was performed well, but there are several concerns that should be addressed by the authors, as following:

*Major concerns*

- In the literatures, there are several systematic reviews that analyzed the nurse-assisted and multidisciplinary out-patients follow-up to patients with liver cirrhosis. This systematic review is missing of novelty.

**Response:**

As presented in the introduction (p.3 l. 77), a recent meta-analysis of four studies found no advantage of self-management programs for patients with liver cirrhosis. We have found reviews dealing with specific tasks in the outpatient follow-up, i.e paracentesis, nutrition, and physical exercise, but nothing on the delivery of these tasks in a nurse-assisted or multidisciplinary out-patient setting.

Due to the low number of patients included in the randomized studies, we believe by the inclusion of non-randomized controlled trials and prospective cohort studies would lead to a more thorough view of the effects of types of interventions and the effects of nurse-assisted and multidisciplinary follow-up after admission with decompensation in patients with liver cirrhosis from all causes.

- Authors did not perform any statistical analysis in the following study.

**Response:**

In this article, we present a systematic review without a meta-analysis. As presented in the main manuscript in the discussion, p. 26, l. 447; Initially, we planned to perform meta-analyses of mortality and readmission outcomes. Due to the heterogeneity of the eight randomized studies concerning populations, interventions, controls, and outcomes, a meta-analysis was not found justified to present in the article.

We have performed the meta-analysis, and the results can be presented as supplementary material if requested by the reviewers.

- In introduction, authors should explain better that there are two stage of liver cirrhosis (compensated and decompensated) as well as two different approaches to care these patients.

**Response:**

We agree and have changed the introduction accordingly.

- Search strategy: authors should explain more clearly (i) which data range they choose, (for instance: all the databases that they use from 1992 to 2022) and (ii) why they choose that range.

**Response:** We did not include a limit on publication dates which is now stated in the Search strategy p. 6 l. 112.

- Result paragraph: data are presented in very descriptive way. They should be more focused on the comparison between groups (controlled trials vs allocated trials) and describe in more detail way what they discovered.

**Response:** We believe that the descriptive overview of the included interventions provides the reader with a perspective on the range and content of the interventions.

We have changed the presentation of the Intervention Outcomes section of our study p. 19 l. 275. The changes provide the reader with a greater overview of the primary endpoints in this review and the results of the specific outcomes in each of the studies. Table 2 further provides a complete overview of intervention endpoints, measurements, and outcomes for the 16 individual studies.

**Reviewer #3:**

Dr. O'Connell and others made a systematic review on "Nurse-assisted and multidisciplinary outpatient follow-up among patients with decompensated liver cirrhosis" by searching the databases Pubmed, Embase, Cinahl, Web of Science, and Cochrane Libraries, 75 Conference proceedings, reference lists, and bibliographies (manually). There are included eleven controlled studies and five cohort studies comprising 1224 participants. The conclusions suggested positive effects of nurse-assisted and multidisciplinary follow-up for outpatients with 44 liver-cirrhosis.

The meta-analysis method applied, the data, and the results look like very interesting. On the contrary, the results were reached based on the multi parameters of different categories, the criteria to classify the data are very confused, and very difficult to be justified to get the conclusions.

There are no quantified data to show the significant differnece for the comparisons among the randomized, non-randomized patients and other groups with and without the nurse-assisted and multidisciplinary outpatient follow-up.

Overall, this meta data analysis must be adjusted to be more accurate, precise, and the paper be more academic readable, no ambiguity.

It is much appreciated if the authors could classify the parameters with some grades/levels and do further statistical analysis and show the solid results. The final solid conclusions are expected for your further analysis if possible.

**Response:** We have now modified the result section in order to present the data more precisely. See answers to reviewer 2.

**Reviewer #4:**

Malene Barfod O’Connell and co-authors have done a systematic review on the highly relevant topic of nurse-assisted and multidisciplinary follow-up among patients with liver cirrhosis. The manuscript is well written and presents the systematic review in a comprehensible way. The systematic review is based on a protocol published prior to the conduct of the review and a PRISMA 2020 checklist is included in the submission.

The authors identified sixteen studies comprising 1224 participants through a comprehensive literature search strategy. The quality of the included studies was low to moderate with mixed results with regards to the interventions impact on outcomes which in turn led to the conclusion that no data currently supports a specific type of nurse-assisted post-discharge intervention.

Furthermore, a pre-planned meta-analysis was not justified due to the heterogeneity of the included randomized studies. In the final conclusion of the manuscript the authors nevertheless state that the review indicated that the nurse-assisted intervention could positively affect the selected objective outcomes. The devil’s advocate might conclude the opposite conclusion.

**Response:** We agree that it is difficult to justify a conclusion on the effect of Nurse-assisted and multidisciplinary outpatient follow-up among patients with decompensated liver cirrhosis. We have adjusted both the discussion, p.24 l. 392, and the conclusion, p. 26, l. 452 to be more accurate.

In an effort to assess the overall quality of the systemic review I have identified a few points listed below. Scoring the systematic review according to the AMSTAR-2 tool (Shea BJ et al. BMJ. 2017 Sep 21;358:j4008) yields a low quality score.

***1. The PROSPERO protocol***

- The PROSPERO record is not matching the methods described in the manuscript (e.g. language restrictions on included manuscripts, the health care personnel considered (nurses vs. physicians) and intervention types).
- Deviations from the protocol should be explained in the manuscript.

**Response:** We have now made the necessary changes to the manuscript, in order to explain the deviations from the protocol.

Since we didn’t exclude any manuscripts based on the language, we have removed the following sentence from the manuscript: “Studies without a full text available in English were excluded.”

The research question stated in the PROSPERO record was: “Do physician or nurse-assisted follow-up interventions after an admission to the hospital with decompensation or complications to liver disease in patients with liver cirrhosis benefit the patients?”. This sentence is miswritten since it was never our intention to include studies with solely physician-driven interventions. We have therefore changed this to “nurse-assisted and multidisciplinary interventions”. In the eligibility criteria, p. 5, l. 88 we further specify this with the following: “ii) if nurse-assisted follow-up, including nurse-assisted multidisciplinary interventions, was described in the manuscript“.

- The PROSPERO record apparently (as of 28-SEP-2022) has not been updated with regards to the stage of the review.

**Response:** The Prospero recorded has now been updated.

***2. Search strategy:***

- It is not described whether the reference lists of the included studies were included in the search

**Response:** In the search strategy, p. 6. l. 111, we state that reference lists were searched manually. We have changed the sentence to be more precise.

- It is not described whether trial registries were included in the manual search

**Response:** We have searched the trial registries. This is now stated in the search strategy p. 6. l. 110.

- I cannot find a list of the excluded studies with an explanation of why they were excluded

**Response:** We have now included the list of excluded studies as supplementary material (supplementary table S2) p. 6 l. 115
